# Effects of 5-Fluorouracil in Nuclear and Cellular Morphology, Proliferation, Cell Cycle, Apoptosis, Cytoskeletal and Caveolar Distribution in Primary Cultures of Smooth Muscle Cells

**DOI:** 10.1371/journal.pone.0063177

**Published:** 2013-04-30

**Authors:** Marcelo de Carvalho Filgueiras, Alexandre Morrot, Pedro Marcos Gomes Soares, Manoel Luis Costa, Cláudia Mermelstein

**Affiliations:** 1 Laboratório de Diferenciação Muscular e Citoesqueleto, Instituto de Ciências Biomédicas, Universidade Federal do Rio de Janeiro, Rio de Janeiro, Brazil; 2 Departamento de Morfologia, Universidade Federal do Ceará, Fortaleza, Ceará, Brazil; 3 Universidade Federal do Piauí, Piauí, Brazil; 4 Laboratório de Imunologia, Instituto de Microbiologia Paulo de Góes, Universidade Federal do Rio de Janeiro, Rio de Janeiro, Brazil; National Cancer Institute, United States of America

## Abstract

Colon cancer is one of the most prevalent types of cancer in the world and is one of the leading causes of cancer death. The anti-metabolite 5- fluorouracil (5-FU) is widely used in the treatment of patients with colon cancer and other cancer types. 5-FU-based chemotherapy has been shown to be very efficient in the improvement of overall survival of the patients and for the eradication of the disease. Unfortunately, common side effects of 5-FU include severe alterations in the motility of the gastrointestinal tissues. Nevertheless, the molecular and cellular effects of 5-FU in smooth muscle cells are poorly understood. Primary smooth muscle cell cultures are an important tool for studies of the biological consequences of 5-FU at the cellular level. The avian gizzard is one of the most robust organs of smooth muscle cells. Here we studied the molecular and cellular effects of the chemotherapic drug 5-FU in a primary culture of chick gizzard smooth muscle cells. We found that treatment of smooth muscle cells with 5-FU inhibits cell proliferation by the arrest of cells in the G1 phase of cell cycle and induce apoptosis. 5-FU induced a decrease in the percentage of histone H3-positive cells. Treatment of cells with 5-FU induced changes in cellular and nuclear morphology, a decrease in the number of stress fibers and a major decrease in the number of caveolin-3 positive cells. Our results suggest that the disorganization of the actin cytoskeleton and the reduction of caveolin-3 expression could explain the alterations in contractility observed in patients treated with 5-FU. These findings might have an impact in the understanding of the cellular effects of 5-FU in smooth muscle tissues and might help the improvement of new therapeutic protocols for the treatment of colon cancer.

## Introduction

Colon cancer is one of the most prevalent types of cancer in the world and is one of the leading causes of cancer death. 5-FU is a commonly used chemotherapy agent in the treatment of human colon cancer [1]. The anti-metabolite 5- fluorouracil (5-FU) is widely used in the treatment of patients with colon cancer and other cancer types. 5-FU-based chemotherapy has been shown to be very efficient in the improvement of overall survival of the patients and for the eradication of the disease. 5-FU is a pyrimidine analogue, that within the cell, is converted into 5-fluoro-2′deoxy-5′monophosphate resulting in the inhibition of thymidylate synthase with the subsequent suppression of DNA synthesis [2]. One major side effect is that 5-FU treatment induces severe alterations in the motility of the gastrointestinal tissues in patients [3], [4]. Soares and colleagues [5] described a gastrointestinal dysmotility in 5-FU-induced intestinal mucositis in rats even 15 days after treatment when the inflammatory process was resolved. They found a delay in gastric emptying in vivo and a significant increase in gastric fundus and duodenum smooth muscle contraction.

Nevertheless, the molecular and cellular effects of 5-FU in smooth muscle cells are poorly understood. Primary smooth muscle cell cultures are an important tool for studies of the biological effects of 5-FU at the cellular level. The major advantages of using *in vitro* cell cultures are the consistency, reproducibility and the possibility of a detailed analysis at both the molecular and cellular levels. Cytoskeletal distribution and cell proliferation are especially suitable for cell culture studies.

Although smooth muscle cells are present in several organs in all vertebrates, the avian gizzard is the most enriched in smooth muscle cells. In the chick gizzard, smooth muscle cells exist among a matrix of connective tissue and extracellular matrix [6]. Smooth muscle is distinguished from cardiac and skeletal muscle because it lacks sarcomeres. Instead, they display long myosin II filaments that slide along actin filaments. These actin filaments are linked to the cell membrane at attachment plaques and within the cell to cytoplasmic dense bodies. In culture, smooth muscle cells display abundant stress fibers, as well as actin based-membrane protrusions. Dense bodies are linked to a dense network of desmin intermediate filaments [7], [8]. The membrane of smooth muscle cells contains interspersed regions of dense bodies and caveolae. Caveolae are flask-shaped invaginations that appear in rows in periodic register along the longitudinal axis of the smooth muscle sarcolemma [9]. The cytoskeleton and caveolae confers upon smooth muscle cells a long and fusiform shape, and the alternate contraction of circular and longitudinal layers of smooth muscle is responsible for digestive motility.

In the present work we studied the effects of the anti-metabolite drug 5- fluorouracil (5-FU) in the overall cellular morphology, cytoskeletal and caveolar organization, cell proliferation and cell death of chick cultured smooth muscle cells. This work may contribute to the understanding of the cellular and molecular mechanisms involved in the alterations in the motility of the gastrointestinal tissues in patients treated 5-FU. Our results could have an impact on the use of alternative therapeutic approaches in the treatment of colon cancer.

## Materials and Methods

### Antibodies and Probes

DNA-binding probe DAPI (4,6-Diamino-2-phenylindole dyhydrochloride) and filamentous-actin specific probe Texas red-phalloidin were purchased from Molecular Probes (USA). Annexin V-FITC was purchased from BD Immunocytometry Systems (USA). Rabbit polyclonal anti-desmin, rabbit polyclonal anti-tropomyosin, and mouse monoclonal anti-alpha-smooth muscle actin (clone 1A4) antibodies were from Sigma Chemical Co. (USA). Rabbit polyclonal anti-phospho-histone H3 was from Upstate Millipore (USA). Mouse monoclonal anti-caveolin-3 (clone 26) was purchased from BD Pharmigen (USA). Alexa Fluor 488-goat anti-mouse/rabbit IgG and Alexa Fluor 546-goat anti-mouse/rabbit IgG antibodies were from Molecular Probes (USA).

### Cell Cultures

This study using chick embryos was approved by the Ethics Committee for Animal Care and Use in Scientific Research from the Federal University of Rio de Janeiro and received the approval number: DAHEICB 004. All cell culture reagents were purchased from Invitrogen (São Paulo, Brazil). Primary cultures of smooth muscle cells were prepared from gizzards of 11-day-old chick embryos [10]. Fragments of gizzard smooth muscle were incubated at 37°C for 15 min in calcium-magnesium-free solution (CMF) containing 0.2% trypsin. Then, the supernatant was gently removed and discarded. The fragments of gizzard muscle were further incubated once at 37°C for 15 min in CMF containing 0.2% trypsin. The suspension containing isolated cells and tissue fragments was centrifuged, and the pellet was dispersed by repeated pipetting in cultured medium (Minimum Essential Medium with 10% horse serum, 0.5% chick embryo extract, 1% L-glutamine and 1% penicillin-streptomycin). The resulting suspension was filtered and cells were plated at an initial density of 2×10^6^ cells/35 mm culture dishes onto 22 mm-Aclar plastic coverslips (Pro-Plastics Inc., USA) previously coated with rat tail collagen. Cells were grown under humidified 5% CO_2_ atmosphere at 37°C. After the first 24 h, cultures were fed with fresh cultured medium.

After the first 24 h of culture, some cells were treated with 5-fluorouracil (5-FU, Eurofarma Laboratórios, Brazil) at the following different final concentrations: 0.01, 0.05, 0.1, 1, 10 and 50 mM). After 24 h of 5-FU treatment, some cultures were washed with fresh culture medium and grown for the next 48 h.

### Cell Viability

Cell viability was measured by the MTT assay. This assay relies on the ability of viable cells to reduce a yellow tetrazolium salt (MTT; Sigma) metabolically to a purple formazan product. This reaction takes place when mitochondrial reductase enzymes are active. Cells were grown in 96-well plates (1×10^4^/200 µl/well). After the first 24 h of culture, cells were treated for the next 24 h with 5-FU at the final concentrations of 0.01, 0.05, 0.1, 1 and 10 mM. Cells were treated with 10 µl of MTT (5 mg/ml in PBS) for 3 h at 37°C and then 200 µl of DMSO was added to dissolve the precipitated formazan and its absorbance was read in ELISA reader at 570 nm.

### Cell Cycle Analysis

Cell cycle analysis and quantification of apoptosis was carried out by flow cytometry. Smooth muscle cells (2×10^6^) were seeded into 35 mm culture dishes and grown in 2 ml of medium (Minimum Essential Medium with the addition of 10% horse serum, 0.5% chick embryo extract, 1% L-glutamine and 1% penicillin-streptomycin), under humidified 5% CO_2_ atmosphere at 37°C. After the first 24 h of culture, cells were treated for the next 24 h with 5-FU at the final concentrations of 0.01, 0.05, 0.1, 1 and 10 mM. Then, cells were collected by trypsinization, fixed with 70% ethanol and stained with propidium iodide (PI, 20 µg/ml, BD Immunocytometry Systems, USA) in PBS containing 0.1% Triton-X-100 and RNAse (10 µg/ml) for 15 min. Data was acquired on a BD FACS Calibur flow cytometer using CellQuest software (BD Immunocytometry Systems, USA). Ten thousand events were analyzed for each sample. Appropriate gating was used to select the single cell population and used on all samples, ensuring that the measurements were made on a standardized cell population.

### Apoptosis Analysis

Apoptosis analysis was carried out by flow cytometry. Smooth muscle cells (2×10^6^) cells were seeded into 35 mm culture dishes and grown in 2 ml of medium (Minimum Essential Medium with the addition of 10% horse serum, 0.5% chick embryo extract, 1% L-glutamine and 1% penicillin-streptomycin), under humidified 5% CO_2_ atmosphere at 37°C. After the first 24 h of culture, cells were treated for the next 24 h with 5-FU at the final concentrations of 0.01, 0.05, 0.1, 1 and 10 mM. To analyze the presence of floating apoptotic cells in the liquid culture medium, 2 ml of conditioned medium from each experimental condition were collected in separated tubes. Adherent cells were collected by trypsinization, counted and 10^6^ cells of each condition were collected by centrifugation. All samples (adherent cells and floating cells) were incubated with Annexin V-FITC (50 µg/ml, BD Immunocytometry Systems, USA) for 15 min at room temperature and in the dark. A negative control was used with cells that were treated in the same way as described above but omitting the anexin V incubation. As a positive control, some cells were treated with 30% ethanol for 5 min at 37°C in order to induce apoptosis. Data was acquired on a BD FACS Calibur flow cytometer using CellQuest software (BD Immunocytometry Systems, USA). Ten thousand events were analyzed for each sample. Appropriate gating was used to select the single cell population and used on all samples, ensuring that the measurements were made on a standardized cell population.

### Immunofluorescence and Digital Image Acquisition

Cells were rinsed with PBS and fixed with 4% paraformaldehyde in PBS for 10 min at room temperature. They were then permeabilized with 0.5% Triton-X 100 in PBS 3 times for 10 min. The same solution was used for all subsequent washing steps. Cells were incubated with primary antibodies for 1 h at 37°C. After incubation, cells were washed for 30 min and incubated with Alexa Fluor-conjugated secondary antibodies for 1 h at 37°C, and nuclei were labeled with DAPI (0.1 µg/ml in 0.9% NaCl).

For filamentous actin staining, cells were rinsed with PBS and fixed with 4% paraformaldehyde in PBS for 10 min at room temperature. They were then permeabilized with 0.5% Triton-X 100 in PBS 3 times for 10 min and washed with PBS for 30 min. Cells were incubated with the F-actin specific probe Texas red-phalloidin (3.3 µM) for 1 h at 37°C. After incubation, cells were washed with PBS for 30 min and incubated with DAPI (0.1 µg/ml in 0.9% NaCl) for nuclear labeling.

Cells were mounted in Prolong Gold solution (Molecular Probes, USA) and examined with an Axiovert 100 microscope (Carl Zeiss, Germany) and images were acquired with an Olympus DP72 digital camera (Olympus, Japan). Control experiments with no primary antibodies showed only a faint background staining (data not shown). Live cultured cells grown in collagen-coated Aclar coverslips were examined and images were acquired in phase contrast microscopy in the same microscope and digital system described above.

### Morphometrical Analysis

DAPI images were acquired as described above and the total number of nuclei per field was quantified using the public domain NIH ImageJ program (develop at the U.S. National Institutes of Health and available on the internet at http://imagej.nih.gov/ij/). Nuclei morphology was analyzed using ImageJ software by the calculation of the major/minor axis ratio (major and minor are the primary and secondary axis of the best fitting ellipse). A total of at least 200 microscopic fields from at least three different cell cultures were analyzed in each assay.

### Stastistical Analysis

Each experiment was performed in four replicates and the results are the means ± s.d. of three independent experiments. All the values were represented as the means ± standard deviation. Statistical analysis was performed with the use of the Mann-Whitney test (control group versus 5-FU treated group). In this regard, a probability (p) value less than 0.05 was considered statistically significant.

## Results and Discussion

**Figure 1:**
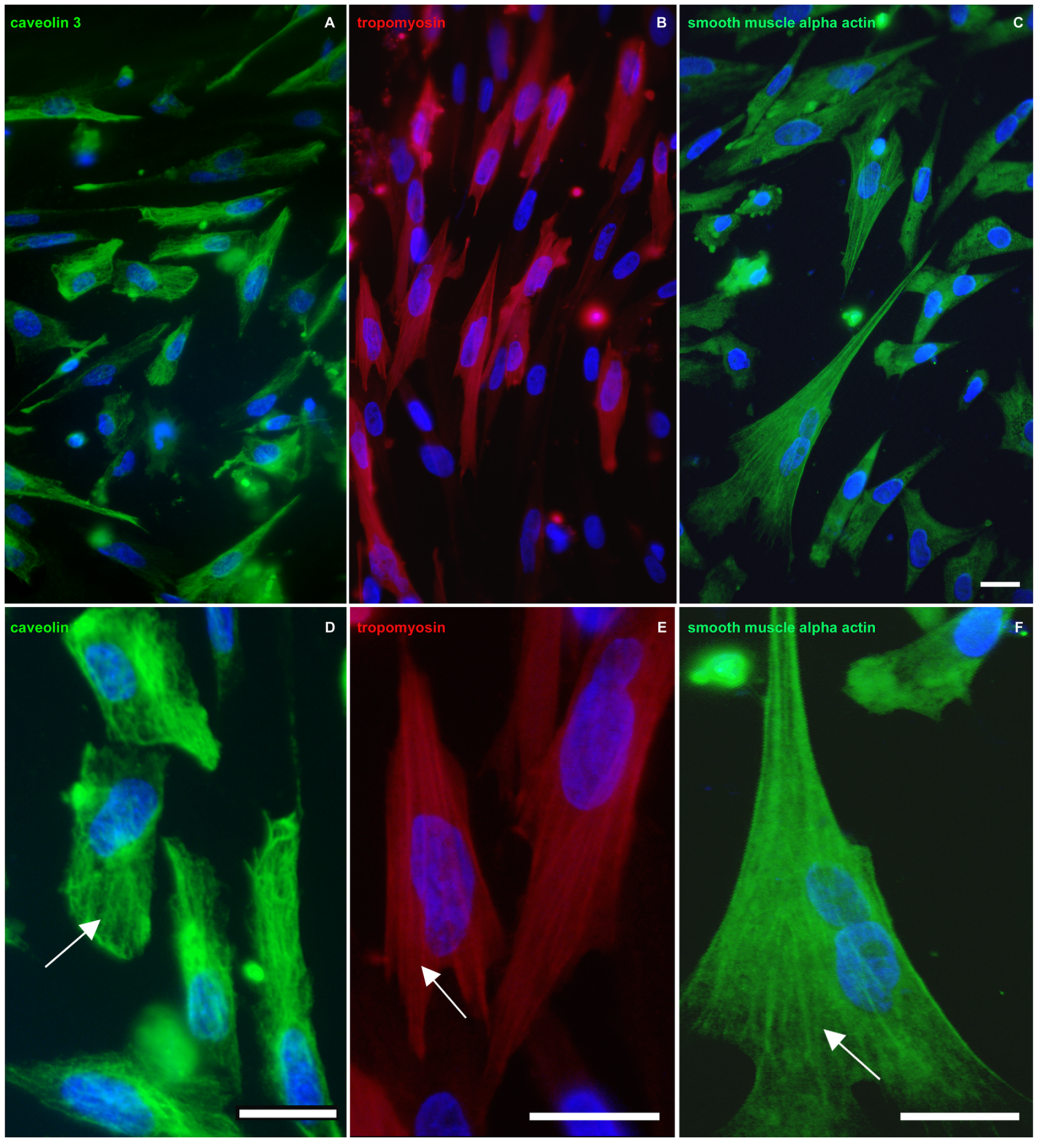
Primary cultures of chick gizzard cells express specific smooth muscle markers. Cells were grown for 24 h, fixed and immunostained for the smooth muscle markers caveolin-3 (green, **A** and **D**), tropomyosin (red, **B** and **E**), and alpha-actin (green, **C** and **F**). Arrow in (**D**) point to a filamentous distribution of caveolin-3, arrows in (**E**) and (**F**) points to stress fibers. Note that the majority of the cells in each field are positive for smooth muscle alpha-actin (**C**), caveolin-3 (**A**), and tropomyosin (**B**). Scale bars = 20 µm.

We used chick gizzard smooth muscle cultures to understand the molecular and cellular effects of 5-FU. Chick gizzard is composed mainly of two types of cells: smooth muscle cells and fibroblasts. In order to characterize the population of cells presents in our primary cultures we analyzed the expression of three smooth muscle specific makers, caveolin-3, tropomyosin and smooth muscle alpha-actin. We found caveolin-3, tropomyosin and smooth muscle alpha actin-positive cells in these chick gizzard cell cultures (**Figure 1**). It is possible to observe the filamentous distribution of caveolin-3 in the subsarcolemmal region of the cells (**Figure 1d**). These results are in agreement with previous reports showing that caveolin-3 is found in filamentous structures longitudinally oriented in chick, human and mouse cultured skeletal muscle cells [11]–[13]. Cultures also displayed well-spread and flattened cells, showing a vast area of attachment to the substrate and a high number of stress fibers (**Figures 1e and 1f**). Quantification of the number of caveolin-3 positive cells showed that 80% of the cells express the muscle specific protein caveolin-3. We also quantified the number of fibroblasts in these cultures by measuring the size/appearance of their nuclei. It has been described that the nuclei of fibroblasts are pale and larger in size when compared with the nuclei of smooth muscle cells isolated from embryonic chick gizzard [**14**]. The nuclei size/appearance quantification showed that 20% of the nuclei are compatible with fibroblasts and 80% are compatible with smooth muscle cells.

**Figure 2:**
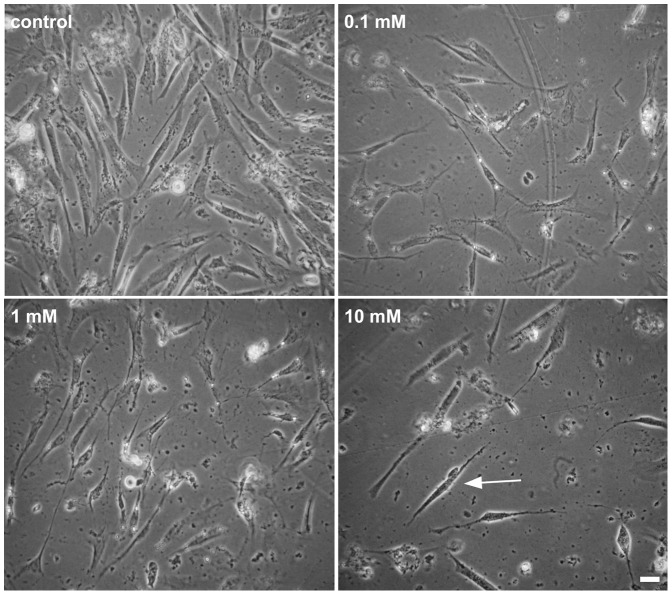
5-FU induces major changes in cell morphology in smooth muscle cell cultures. Phase contrast microscopy shows that cells treated with 1 or 10 mM of 5-FU (C-H) display a spindle-shaped morphology as compared to untreated cells (A and B). Note the decrease in the number of cells per field in cultures treated with 1 and 10 mM of 5-FU. Scale bar = 20 µm.

Having characterized the smooth muscle culture properly, we analyzed the effects of the chemotherapic drug 5-fluorouracil (5-FU) in the cells. 5-FU is one of the most used anti-colon cancer drugs and patients treated with 5-FU display a dysfunction of intestinal motility even weeks after withdrawal of the drug. These persistent drug effects could be related to either the overall smooth muscle cells number or to their structure and physiology. To study the effects of 5-FU in the proliferation of smooth muscle cells, cultures were grown for 24 h and treated for the next 24 h with the following different final concentrations of 5-FU: 0.01, 0.05, 0.1, 1 and 10 mM. Cells were analyzed under phase contrast microscopy (**Figure 2**). Treatment with 5-FU at 0.01, 0.05, 0.1 mM concentrations did not induce visible changes in the overall morphology of cells (data not shown), whereas cells treated with 1 or 10 mM of 5-FU showed a spindle-shaped morphology as compared to untreated cells (**Figure 2**). It is possible to observe a decrease in the number of cells per field in cultures treated with 1 and 10 mM of 5-FU (**Figure 2**).

**Figure 3:**
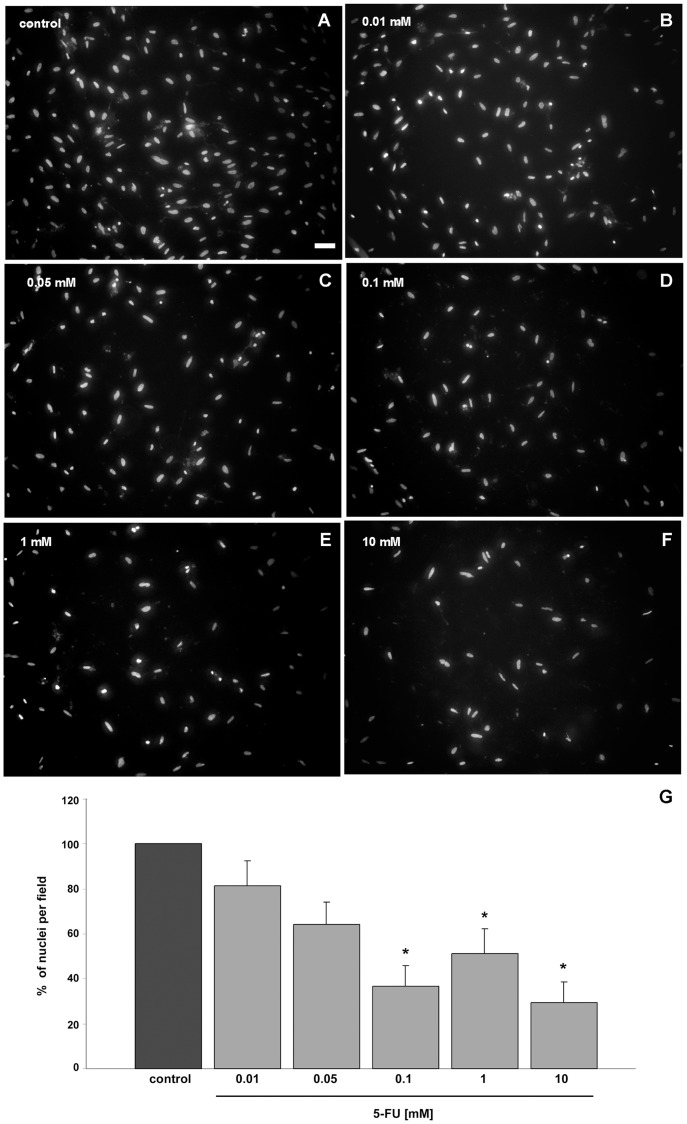
5-FU induces a decrease in the total cell number in cultures of smooth muscle cells. To quantify the change in cell number induced by 5-FU, control (**A**) and 5-FU treated-cells (**B–F**) were stained with the nuclear dye DAPI. Quantification of the number of nuclei per field revealed that 5-FU treatment induced a decrease in cell number in all the concentrations tested (**G**). We found an initial reduction of 20% at 0.01 mM up to a reduction of 70% in 10 mM of 5-FU. At least 50 microscopic fields for each culture condition were scored in at least three independent experiments. Values were represented as the means ± standard deviation. Statistical analysis was performed by P<0.05 Mann-Whitney test (control group versus 5-FU treated group). Scale bar = 50 µm.

To further evaluate the effects of 5-FU in the total number of cells present in the smooth muscle cultures, untreated and 5-FU treated cells were fixed with 4% paraformaldehyde and stained with the DNA specific probe DAPI (**Figure 3**). 5-FU treatment induced a decrease in the number of nuclei per microscope field in all the concentrations tested. We observed a significant dose-response, with initial reduction of 20% at 0.01 mM 5-FU up to a reduction of 70% in 10 mM (**Figure 3**). These results suggest that 5-FU inhibits the proliferation of chick smooth muscle cells grown in culture. It is important to point out that 5-FU doses used for human cancer treatment are in the range of 12–15 mg per kg, which corresponds to a concentration of 0.1 mM of 5-FU (assuming that 1 kg of human body weight corresponds to a 1 liter volume). In the present study we found a 60% reduction in the number of smooth muscle cells after 24 hours of treatment with 0.1 mM of 5-FU.

**Figure 4:**
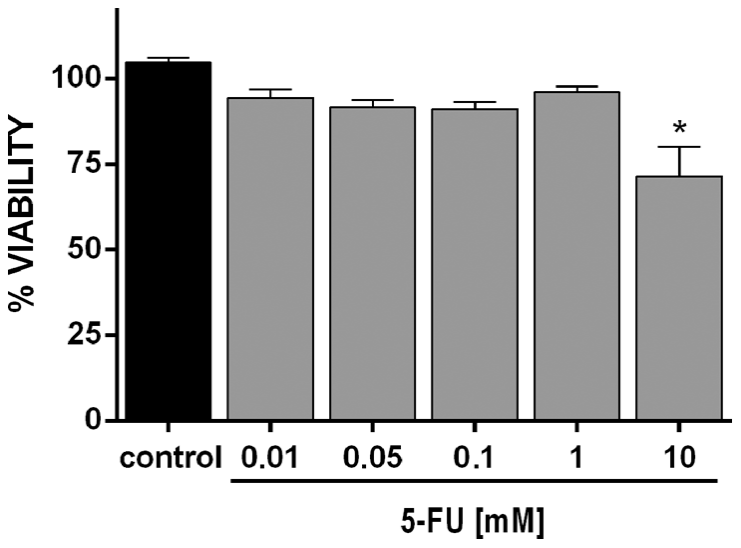
Cell viability is reduced after 5-FU treatment in smooth muscle cell cultures. A MTT-based method was used to analyze cell viability in untreated and 5FU-treated smooth muscle cell cultures. Data represent mean absorbance minus background from triplicate wells. Note the 30% reduction on cell viability observed after 24 h of treatment with 10 mM of 5-FU. Statistical analysis was performed by P<0.05 Mann-Whitney test (control group versus 5-FU treated group).

To determine whether 5-FU has any effect on cell viability, experiments were done with untreated and treated cell cultures grown for 24 h in the presence of 5-FU, using a MTT-based method. We found a 30% reduction on cell viability in the smooth muscle cell cultures after 24 h of treatment with 10 mM of 5-FU when compared to untreated cells (**Figure 4**). This reduction in cell viability is in accordance with the reduction in the total number of cells observed after treatment with 10 mM of 5-FU (shown in Figure 2).

**Figure 5:**
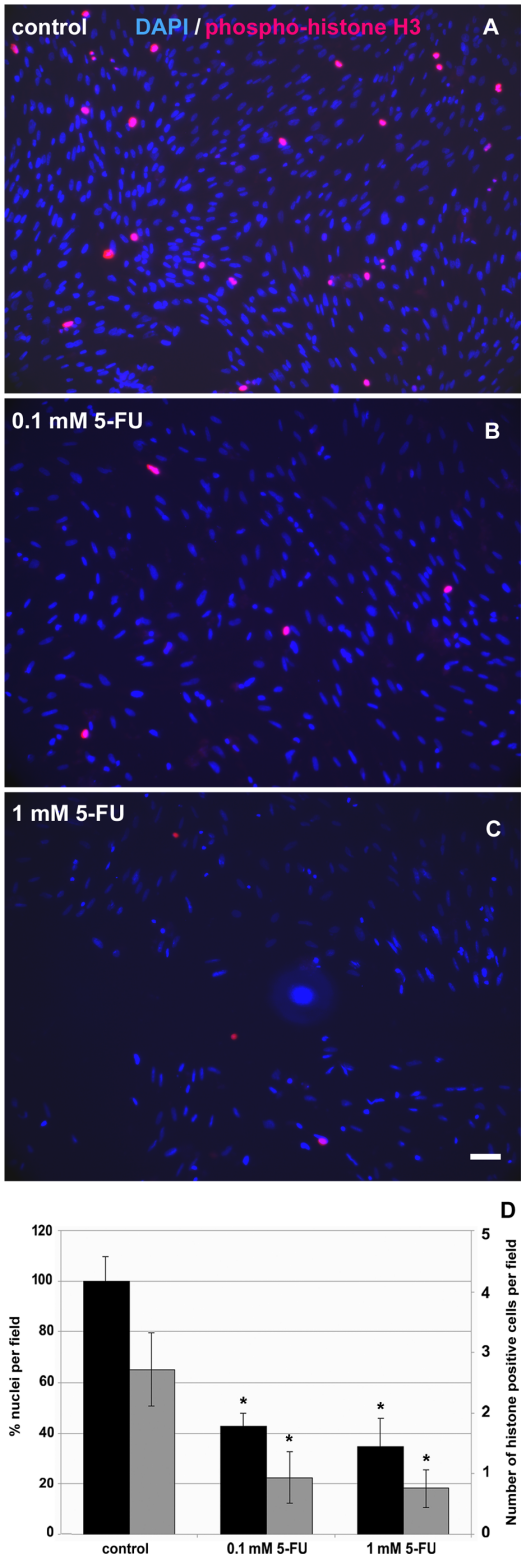
5-FU inhibits cell proliferation in cultures of smooth muscle cells. Cells were grown for 24 h, treated with 5-FU and stained with an anti-phospho-histone H3 antibody (red) and with DAPI (blue). (**A**) control cells, (**B**) 0.1 mM 5-FU and (**C**) 1 mM 5-FU. (**D**) Quantification of the percentage of nuclei/field (black bars) and the number of phospho-histone H3-positive cells/field (gray bars) in each culture condition revealed a 40% decrease in phospho-histone H3-positive cells after 24 h of both 0.1 and 1 mM of 5-FU treatment. At least 50 microscopic fields for each culture condition were scored in at least three independent experiments. Values were represented as the means ± standard deviation. Statistical analysis was performed by P<0.05 Mann-Whitney test (control group versus 5-FU treated group). Scale bar = 50 µm.

Since we found a reduction in the number of cells after 5-FU treatment (**Figure 5**), we decided to test a possible inhibition of proliferation by 5-FU by the analysis of the expression of phospho-histone H3. Phospho-histone H3 is a cellular marker for cell proliferation, since histone H3 is specifically phosphorylated during mitosis. Cells were treated with 0.1 and 1 mM of 5-FU and stained with an antibody against the phospho-histone H3 protein (**Figure 5**). By the quantification of the percentage of phospho-histone H3-positive cells in each culture condition, we estimated a 40% decrease in phospho-histone H3-positive cells after 24 h of both 0.1 and 1 mM of 5-FU treatment. These results, in conjunction with the data shown in **Figure 3**, show that 5-FU inhibits the proliferation of chick smooth muscle cells. These results are in accordance with previous work showing the anti-proliferative effects of 5-FU in smooth muscle cells [15]. Nevertheless, this is the first description of these phenomena in chick smooth muscle cell cultures. We also tested the recover of cells from 5-FU treatment. After 24 hours of 10 mM of 5-FU treatment, cells were grown without the drug for the next 48 hours and cell proliferation was analyzed. Labeling with anti-phosphohistone H3 antibody showed no recover in cell proliferation.

**Figure 6:**
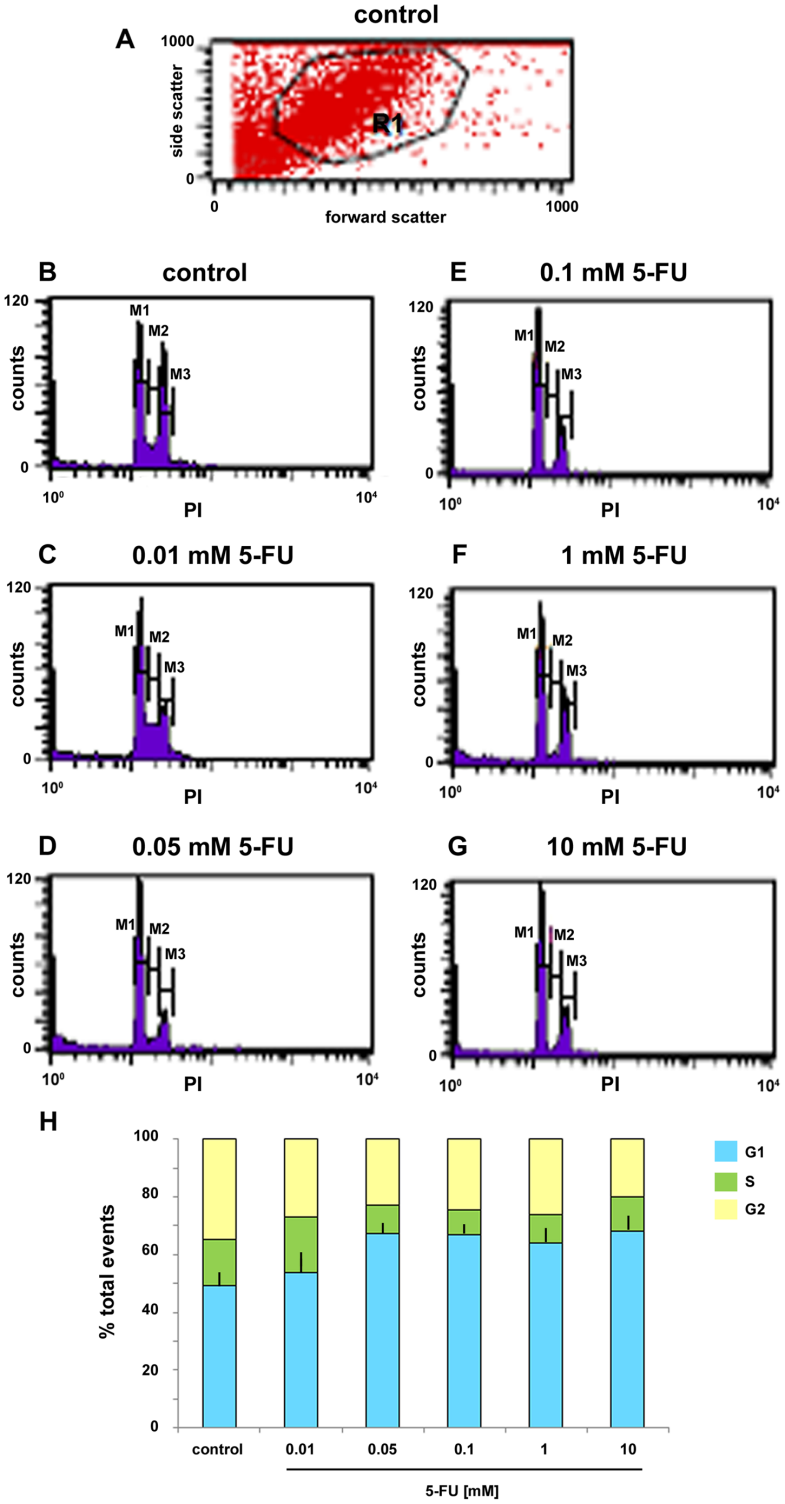
5-FU blocks cell cycle progression in G1 phase. Cell cycle analysis of control (**A** and **B**) and 5-FU treated cells (**C-G**) was evaluated by flow cytometry based on propidium iodide (PI) intercalation into the cellular chromatin (for details see Materials and methods). Data are presented as relative fluorescence intensity. (**H**) Cell sorter analysis revealed that the population of cells in the S and G2 phase was remarkably decreased by 5-FU. Values were represented as the means ± standard deviation.

While several mechanisms are known to interfere with cell proliferation, they act in different cell cycle phases. We decided to analyze the effects of 5-FU in specific cell cycle phases (**Figure 6**). Cells were cultured for 24 h and treated for the next 24 h with 5-FU at the final concentrations of 0.01, 0.05, 0.1, 1 and 10 mM. Then cells were collected by trypsinization, fixed and stained with propidium iodide, which binds specifically to DNA. Cell sorter analysis revealed that the population of cells in the S phase was remarkably decreased by 5-FU (9% of cells in the S phase of the cell cycle in 0.1 mM of 5-FU compared with 16% of control cells, **Figure 6**). Further, the population of cells in the G2 phase was also decreased by 5-FU in all the concentrations tested (20% of cells in the G2 phase of the cell cycle in 10 mM of 5-FU compared with 35% of control cells, **Figure 6**). Interestingly, the population of cells in the G1 phase increased by 5-FU treatment (68% of cells in the G1 phase of the cell cycle in 10 mM of 5-FU compared with 49% of control cells, **Figure 6**). These results indicate that 5-FU inhibits cell proliferation by the induction of cell cycle arrest in G1 phase. Our results with chick smooth muscle cells are in accordance with the data shown by Matuo and colleagues [16] where they show that 5-FU induces an arrest in G1/S phase in the human SW620 colon adenocarcinoma cell line. This is in disagreement with Huang and colleagues [17], who have shown that 5-FU caused a G2/M phase arrest in human keloid fibroblasts. The differential effect of 5-FU in different cell cycle phases could to be related to the cell type and further experiments are needed in order to understand the molecular basis that regulate this process in each of these cells.

**Figure 7:**
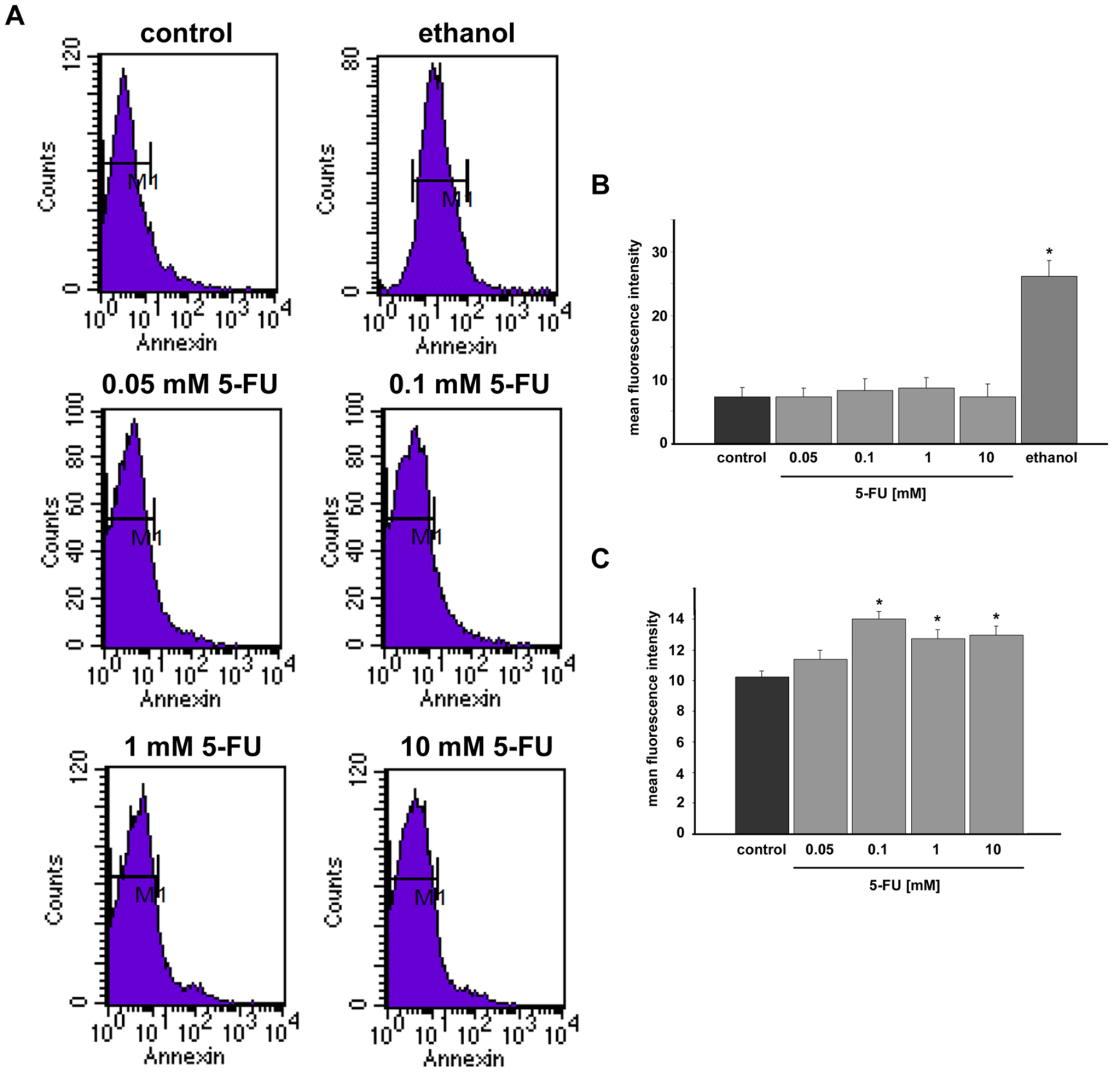
5-FU induces apoptosis in smooth muscle cultures. (**A**) Adherent cells were labeled with annexin V-FITC and analyzed by FACS (for details see Materials and methods). (**B**) Quantification of cell sorter analysis of adherent cells revealing that 5-FU does not induce apoptosis in cells that are attached to the culture dish. (C) Quantification of cell sorter analysis of floating cells revealing 5-FU induction of apoptosis in detached cells. Data are presented as mean fluorescence intensity. As a positive control, some cells were treated with 30% ethanol for 5 min at 37°C in order to induce apoptosis. Values were represented as the means ± standard deviation. Statistical analysis was performed by P<0.05 Mann-Whitney test (control group versus 5-FU treated group).

Then, we decided to analyze whether 5-FU could be inducing apoptosis of smooth muscle cells. Induction of apoptosis was observed in cells incubated with annexin V, which measures the transfer of the phospholipid phosphatidylserine from the inner to the outer membrane of cells and can detect both early and late apoptotic cells with fluorescence cell sorter analysis (**Figure 7**). We analyzed the presence of apoptotic cells in two compartments of the cell cultures: in the cells that were adherent to the culture dish and in floating cells (that were present in the liquid culture medium). After treatment with 5-FU for 24 h at the final concentrations of 0.05, 0.1, 1 and 10 mM, we did not observe apoptosis in adherent cells from all conditions tested (**Figures 7a and 7b**). Importantly, floating apoptotic cells were found in a significant higher amount in the liquid culture medium collected from 0.1, 1 and 10 mM 5-FU treatments, as compared to the medium of untreated cultures. These results indicate that 5-FU, at the concentrations 0.1, 1 and 10 mM and after 24 h of treatment, induces apoptosis in cultured chick smooth muscle cells and the apoptotic cells detach from the culture dish. These results are in agreement with previous data in the literature showing that apoptosis is induced in colon carcinoma cells after 1–3 days of treatment with 5-FU [**18**].

**Figure 8:**
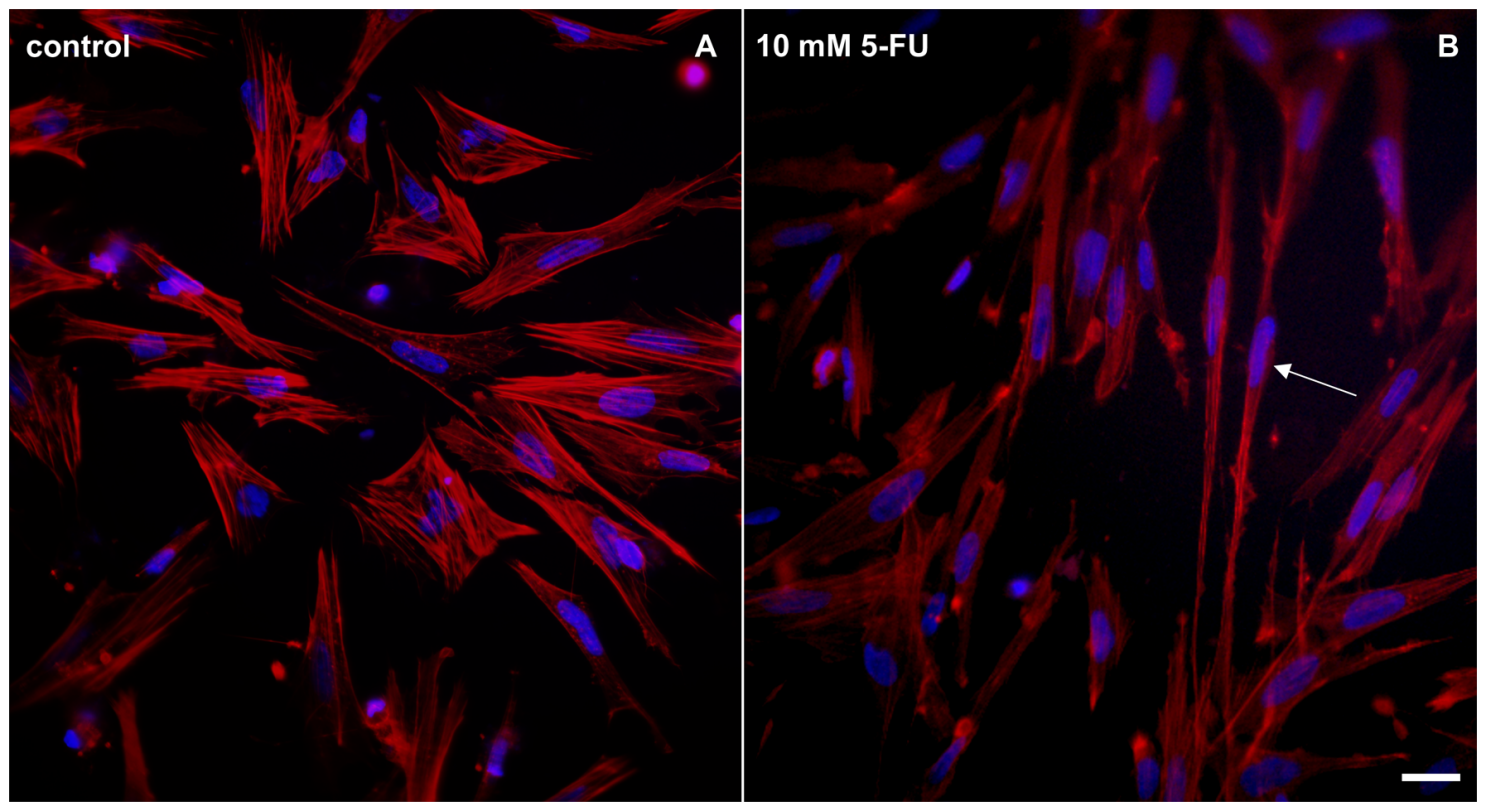
5-FU reduces the number of stress fibers in cultures of smooth muscle cells. Control (**A**) and 5-FU treated-cells (**B**) were stained with Texas red-phalloidin (red) and with DAPI (blue). Note that the majority of untreated cells display actin containing-stress fibers, while cells treated with 10mM of 5-FU show a diminished number of stress fibers in their cytoplasm. 5-FU treated cells show fine membrane projections that are rich in F-actin (arrow in **B**). Scale bar represents 20 µm.

Besides the effects in cell proliferation, we also analyzed changes in the cytoskeleton after 5-FU treatment (**Figure 5**). Since it is well known that the cytoskeleton is one of the major inducers of cell shape, we decided to study the distribution of microfilaments in 5-FU treated cells. Cells were treated with 10 mM of 5-FU and stained with the F-actin specific probe phalloidin (**Figure 8**). 5-FU induced a change in the overall cell shape where cells display long membrane extensions (**Figure 8b**). F-actin labeling also showed the presence of a high number of stress fibers in control cells, while 5-FU induced a reduction in the number of stress fibers within the cytoplasm. Gordon and colleagues [19] showed that 5-FU induces disruption of F-actin containing stress fibers in organ cultures of rat corneal endothelium. So, while our data shows FU-induced alterations in microfilaments in cells *in vitro*, their results show 5-FU affecting microfilaments in cells *in situ* that adhere to a basement membrane.

**Figure 9:**
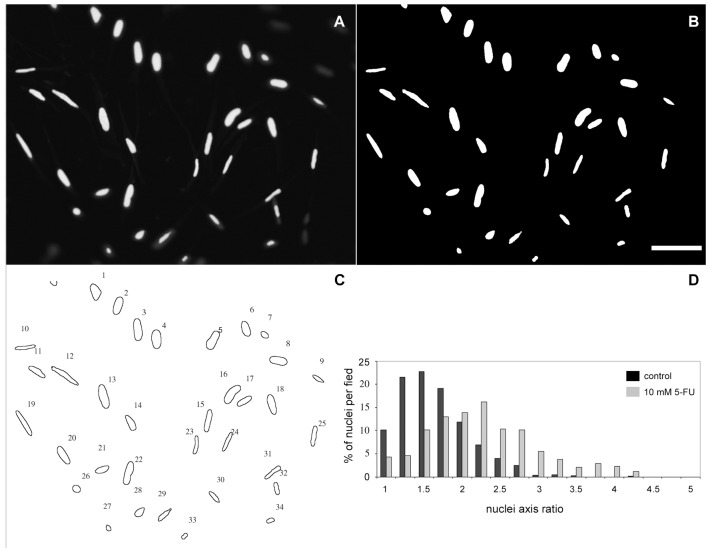
5-FU induces changes in the nuclear shape in cultures of smooth muscle cells. Nuclei morphology was analyzed by the calculation of the major/minor axis ratio of DAPI stained cells. One example of a cell culture treated with 10 mM of 5-FU, stained with DAPI and analyzed for the total number of nuclei per field is shown in images (A–C). (A) raw image of DAPI staining, (B) digitally processed image, (C) nuclei counting. (D) Quantification of the percentage of nuclei/field in control and 5-FU treated cells (10 mM) revealed that more than 70% of control cells have a major/minor axis between 1 and 1.5 µm, while only 40% of 5-FU treated cells have a major/minor axis between 1 and 1.5 µm. At least 200 microscopic fields for each culture condition were analyzed in at least three independent experiments. Values were represented as the means ± standard deviation. Scale bar = 20 µm.

The nuclear labeling of cells showed us that the treatment of cells with 5-FU was inducing a change in the nuclei shape. To further study this phenomenon, we analyzed the shape of the nuclei so in untreated and treated cultures (with 10 mM of 5-FU) by the quantification of the major/minor nuclei axis (**Figure 9**). Measurements of major/minor axis close to 1 µm correspond to circular-shaped nuclei, whereas higher major/minor axis measurements correspond to long and thin-shaped nuclei. We found that 50% of control cells have a major/minor axis between 1 and 1.5 µm, while only 19% of 5-FU treated cells have a major/minor axis between 1 and 1.5 µm. Interestingly, more than 60% of 5-FU treated cells display nuclei with major/minor axis between 2 and 4.5 µm, compared with only 25% of controls cells within this range of nuclei shape. These results show that 5-FU induces major changes in the nuclear shape of smooth muscle cells. Nuclear shape is dependent on cytoskeletal organization and changes in nuclear shape are related to cell differentiation.

**Figure 10:**
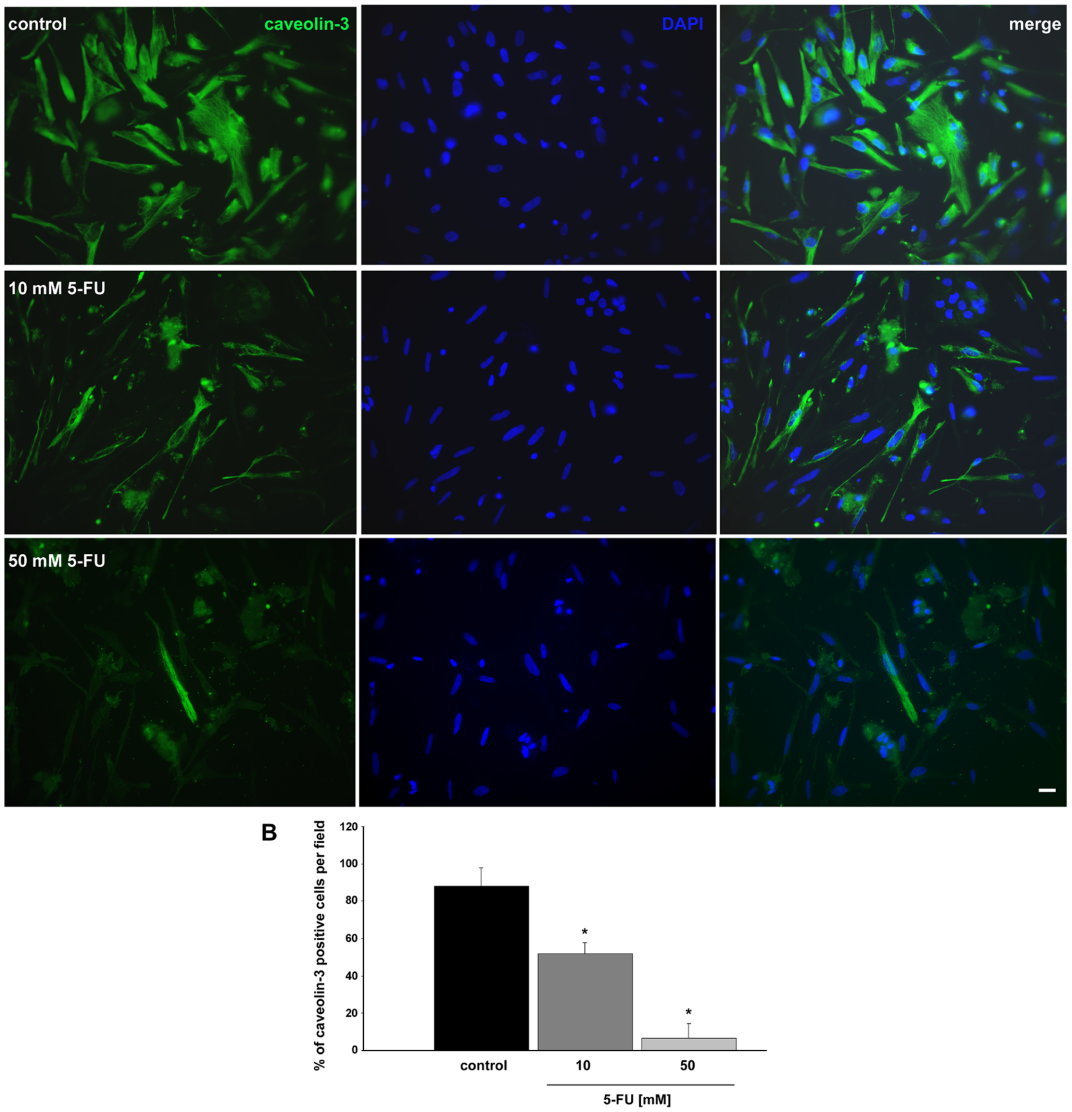
5-FU reduces the expression of caveolin-3 in cultures of smooth muscle cells. Cells were stained with an antibody against caveolin-3 (green) and with DAPI (blue). (**A**) control cells (top images), 10 mM 5-FU (middle images), 50 mM 5-FU (bottom images). Merged images are shown at the right of each panel. (**B**) Quantification of the percentage of caveolin-3 positive cells/field revealed that more than 80% of control cells were positive for caveolin-3, while 5-FU treated cells show a diminished number of caveolin-3 positive cells (50% in 10 mM of 5-FU and 5% in 50 mM of 5-FU). Values were represented as the means ± standard deviation. Scale bar = 20 µm. Statistical analysis was performed by P<0.05 Mann-Whitney test (control group versus 5-FU treated group).

It has been shown that smooth muscle cells are very rich in caveolar structures in their sarcolemma. Caveolae are invaginations of the membrane that concentrate signaling molecules and are characterized by the presence of caveolin proteins. There are three caveolins (1, 2 and 3) and caveolin-3 is muscle specific [20]. Caveolae have been shown to be associated with the cytoskeleton in muscle cells, particularly with intermediate filaments [11]. To investigate whether treatment with 5-FU could interfere with the presence and/or distribution of caveolar structures in chick smooth muscle cells, we analyzed the distribution of caveolin-3 in untreated and 5-FU treated cells. Cells were treated with 10 mM of 5-FU and stained with an antibody against caveolin-3 (**Figure 10a**). Control cells displayed a large number of caveolin-3 positive cells with a filamentous distribution of caveolin-3 at the subsarcolemmal region. Interestingly, we observed a 60% reduction in caveolin-3 positive cells after treatment with 10 mM of 5-FU (**Figure 10b**). Then, we decided to test a higher dose of 5-FU in order to analyze its effects in the expression of caveolin-3 in smooth muscle cells. We observed a 90% reduction in caveolin-3 positive cells after treatment with 50 mM of 5-FU (**Figure 10b**). Our results show that 5-FU induces an important reduction in the expression of caveolin-3 in chick gizzard smooth muscle cultures. This reduction in the expression of caveolin-3 could explain the alterations in contractility observed in patients treated with 5-FU [3], [4]. Caveolae has been shown to be involved in the integration of extracellular contractile signals and intracellular effectors in smooth muscle cells, thus offering an efficient mechanism for smooth muscle excitation-contraction coupling [9].

Here we show that treatment of chick gizzard smooth muscle cells with the anti-metabolite 5-FU inhibits cell proliferation by the induction of cell cycle arrest in G1 phase, induces apoptosis, promotes changes in the cellular and nuclear morphology, induces a reduction in the number of actin containing-stress fibers, and induces an important reduction in the number of caveolin-3 positive cells. 5-FU is a commonly used chemotherapy agent in the treatment of human colon cancer, while little is known about the cellular basis of its secondary effects on gastrointestinal dismotility observed in cancer treated patients. Our results suggest that the disorganization of the actin cytoskeleton and the reduction of caveolin-3 expression could explain the alterations in contractility observed in patients treated with 5-FU. These results could be an important starting point towards the understanding of the molecular and cellular effects of 5-FU treatment in smooth muscle cells and might help the development of new therapeutic strategies directed to the improvement of colon cancer treatments.
